# Impact of Cigarette Smoking on Tear Function and Correlation between Conjunctival Goblet Cells and Tear MUC5AC Concentration in Office Workers

**DOI:** 10.1038/srep27699

**Published:** 2016-06-14

**Authors:** Yuichi Uchino, Miki Uchino, Norihiko Yokoi, Murat Dogru, Motoko Kawashima, Aoi Komuro, Yukiko Sonomura, Hiroaki Kato, Pablo Argüeso, Shigeru Kinoshita, Kazuo Tsubota

**Affiliations:** 1 Department of Ophthalmology, Keio University School of Medicine, Tokyo, Japan; 2 Ryogoku Eye Clinic, Tokyo, Japan; 3 Department of Ophthalmology, Kyoto Prefectural University of Medicine, Kyoto, Japan; 4 Schepens Eye Research Institute and Massachusetts Eye and Ear, Department of Ophthalmology, Harvard Medical School, Boston, Massachusetts USA

## Abstract

The first aim of this study was to clarify whether cigarette smoking affects tear secretion, goblet cell density, and tear MUC5AC concentration. The second purpose was to evaluate the correlations of conjunctival goblet cell density with tear MUC5AC concentration and other ocular surface evaluation factors. This cross-sectional study included 88 office workers. All subjects were required to fill in dry eye and smoking questionnaires, in addition to ocular surface evaluation. Tear wash fluid was collected from inferior fornix, and conjunctival epithelium was obtained by impression cytology. Tear MUC5AC concentration was quantified using enzyme-linked immunoassay, and conjunctival goblet cell density was counted after Periodic-acid Schiff staining. Tear MUC5AC concentration had significant positive correlation with conjunctival goblet cell density (r = 0.181, P = 0.03). In current smokers, Schirmer I test value, goblet cell density and tear MUC5AC concentration were significantly lower than non-smokers. Pack-years of smoking have significant negative correlation with goblet cell density (r = −0.174, P = 0.036) and tear MUC5AC concentration (r = −0.183, P = 0.028). We concluded that smoking might decrease tear secretion, goblet cell density and tear MUC5AC concentration. In addition, MUC5AC concentration in tears depends on goblet cell density in the conjunctiva among office workers.

The ocular surface is covered by a tear film for lubrication, playing the role of a nutritional route for corneal epithelium, protecting against pathogens, and maintaining the smooth refractive surface of the eye for vision^1^,^2^. The tear film contains many substances secreted by different glands and tissues around the ocular surface, and is composed of multiple layers. The outer layer of the tear film consists of lipids secreted from the meibomian glands to slow evaporation of aqueous components in tears^3^. The intermediate aqueous layer includes electrolytes and water from the lacrimal glands, as well as gel-forming mucin, primarily MUC5AC secreted by the goblet cells of the conjunctiva^4^,^5^. Tear MUC5AC has an important barrier function, providing hydration and lubrication of the corneal and conjunctival epithelial surfaces, with a 40-MDa molecular weight and glycosylation of large central tandem repeat domain flanked by cysteine-rich domains^2^,^6^. The MUC5AC secretion from conjunctival goblet cells is regulated by parasympathetic and sympathetic nerves^7^,^8^; the parasympathetic neurotransmitters acetylcholine and vasoactive intestinal peptide (VIP), in particular, stimulate goblet cell secretion^9^,^10^. The MUC5AC concentration in tears of dry eye patients is lower than that in normal subjects^11^,^12^. Goblet cell density is also lower in severe dry eye diseases, including ocular cicatricial pemphigoid (OCP)^13^, Stevens−Johnson syndrome, and alkali burn^14^. Hence, it has been thought that the concentration of MUC5AC in tears may depend on the number of goblet cells and/or their physiological condition. However, it remains unclear whether the secreted MUC5AC concentration in tear is significantly correlated with goblet cell density in the human conjunctiva.

The ocular surface of indoor workers is at risk of increased tear evaporation due to low humidity, high room temperature^15^, decreased blinking rate^16^, and indoor pollution including exposure to propylene glycol^17^ used as water based paints and cooling liquids, and volatile organic compounds (VOCs) used in oil based paints, dry cleaning products, and varnishes of the indoor office^18^. Cigarette smoking is also an indoor pollutant, and induces a high prevalence of eye complaints as compared to non-smokers^19^. Cigarette smoke is a well-known, significant source of toxic minerals and heavy metals, including more than 4000 toxic chemical substances^20^. Epidemiological studies have shown that cigarette smoking may be a high-risk factor for several ophthalmological disorders, including cataract^21^, age-related macular degeneration^22^, and dry eye disease^23^. Matsumoto *et al*. have shown significant loss of goblet cells and squamous metaplasia in smokers^24^. Furthermore, tear break-up time (TBUT) is lower among chronic smokers than among non-smokers^25^. However, it has not been clarified whether the MUC5AC concentration in the tears of smokers was significantly decreased compared to that in non-smokers.

This study aimed to clarify the relationship between cigarette smoking and tear secretion, goblet cell density in the conjunctiva, and tear MUC5AC concentration. We also investigated the correlation between goblet cell density and MUC5AC concentration in tears.

## Results

### Participant Characteristics

Participant characteristics and demographic data are reported in Table 1. The mean participant age was 41.8 ± 9.9 years; 44.3 ± 10.1 years for men, and 37.4 ± 7.7 years for women. Subjects between 30 and 49 years of age constituted 60% of the total study population. The mean duration of visual display terminal (VDT) use was 6.8 ± 1.9 h per day: 6.3 ± 1.9 h for men and 7.5 ± 1.7 h for women. Sixteen men reported to be current smokers. Subjects who had undergone ophthalmic surgery constituted 10.2% of the study population.

### Evaluation of Tear Condition Factors in Categorized Groups

The average TBUT was 4.3 ± 2.7 s; 4.5 ± 3.0 s in men and 3.9 ± 2.1 s in women. There was no significant difference in the average TBUT among the four age groups (1; 20–29 years, 2; 30–39 years, 3; 40–49 years and 4; 50 years and over; Table 2). The mean Schirmer test value was 18.0 ± 11.8 mm in eyes of all subjects; 16.6 ± 11.8 mm among men and 20.7 ± 11.5 mm among women. The mean Schirmer test value in subjects over 50 years of age (12.5 ± 9.1 mm) was significantly lower than that in subjects in their 20 s (24.6 ± 11.1 mm, P = 0.001). Likewise, the average Schirmer value in those who did not use contact lenses (CL) (16.5 ± 11.7 mm) was lower than that in CL users (20.9 ± 11.6 mm, P = 0.041). The mean Schirmer test value in current smokers (13.3 ± 11.5 mm) was significantly lower than that in non-smokers (19.0 ± 11.7 mm, P = 0.016; Table 2).

### Goblet Cell Density and Tear MUC5AC Concentration in Categorized Groups

The mean goblet cell density was 97.9 ± 99.1 cells/mm^2^ (median = 66.1 cells/mm^2^) in 145 eyes. The average goblet cell density was 93.1 ± 93.3 cells/mm^2^ in men and 107.2 ± 109.7 cells/mm^2^ in women (Table 3); these values did not differ statistically significantly between men and women (P = 0.333), or among the four age groups (P = 0.387). The mean goblet cell density in current smokers (57.6 ± 30.8 cells/mm^2^) was significantly decreased compared to non-smokers (106.7 ± 106.5 cells/mm^2^, P = 0.036). The mean MUC5AC concentration in the tear fluid was 7.0 ± 8.7 ng/mg in the 145 eyes; this value was 6.8 ± 8.8 ng/mg in men and 7.4 ± 8.6 ng/mg in women (Table 3), which was not statistically significantly different (P = 0.891). However, the average tear MUC5AC concentration in the elderly group in their 50 s (11.4 ± 13.1 ng/mg) was significantly higher than that in subjects in their 20 s or younger (4.4 ± 3.3 ng/mg, P = 0.016). We also found that current smokers had significantly lower MUC5AC concentration (4.1 ± 3.9 ng/mg) than non-smokers (7.6 ± 9.3 ng/mg, P = 0.024). Multiple regression analysis showed that current smoking status had a significantly negative correlation with goblet cell density (P = 0.026) and tear MUC5AC concentration (P = 0.015; Table 4). Furthermore, 10-year increases in age had a significantly positive correlation with tear MUC5AC concentration (P = 0.002), but no significant correlation with goblet cell density.

### Correlation of Cigarette Smoking History with Goblet Cell Density and Tear Mucin Concentration

**Figure 1:**
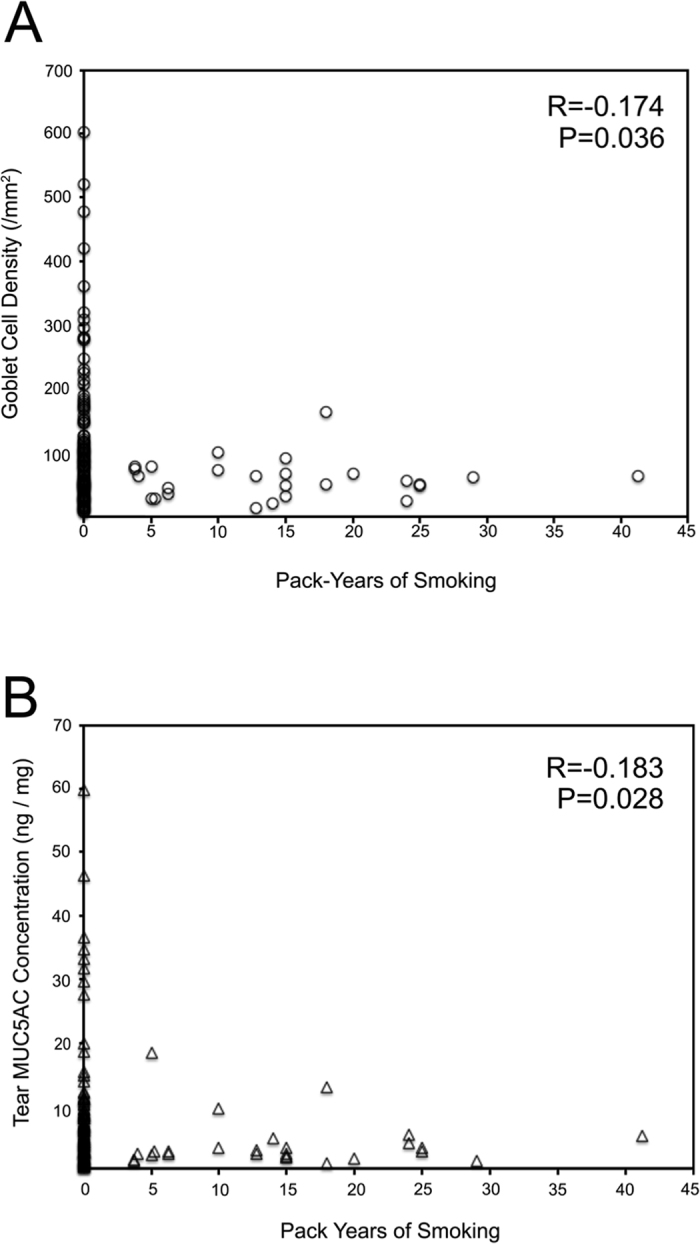
Correlation of pack-years of smoking with goblet cell density and tear MUC5AC protein concentration. Pack-years of cigarette smoking was significantly inversely correlated with goblet cell density and MUC5AC protein concentration in tears.

Pack-years of cigarette smoking was significantly inversely correlated with goblet cell density (r = −0.174, P = 0.036, Fig. 1A) as well as with MUC5AC protein concentration in tears (r = −0.183, P = 0.028, Fig. 1B).

### Correlation between Goblet Cell Density and Ocular Evaluation Factors

Goblet cell density showed a significant, positive correlation with MUC5AC protein concentration (r = 0.181, P = 0.030) and a significant inverse correlation with the lissamine green (LG) staining score (r = −0.185, P = 0.026, Table 5). There was no significant correlation of goblet cell density with the Schirmer test value, TBUT, or FL staining score.

## Discussion

Goblet cells are the main source of the secreted gel-forming mucin MUC5AC in the tear film. The reported goblet cell density values vary from 24 to 2226 cells/mm^2^ under normal conditions^26^. It has been reported that goblet cell number increases from the superior temporal region to the inferior nasal region when the tear drainage system is present^27^. In this study, we collected impression cytology samples from the superior temporal location of the conjunctiva, which was reported to have the lowest goblet cell density area in the ocular surface^2^,^27^. We detected an average goblet cell density of 97.9 ± 99.1 cells/mm^2^ in the superior temporal bulbar conjunctiva of all subjects. It has previously been shown that patients with severe ocular conditions, such as Sjögren syndrome, OCP, Stevens−Johnson syndrome and alkali burn, had a significantly lower goblet cell density, ranging from 0.3 cells/mm^2^ to 26.3 cells/mm^2^, than normal subjects^13^,^14^,^28^. Goblet cell density in patients with Sjögren syndrome is decreased to 12% compared to that in normal subjects^28^, to 10% in Stevens−Johnson syndrome, and 3–5% in OCP^13^,^14^. However, these papers did not discuss the correlation between the tear MUC5AC protein concentration and goblet cell density.

Another report had previously shown the correlation between goblet cell density and *MUC5AC* gene expression level only^29^. In our study, we found that goblet cell density has a significantly positive correlation with MUC5AC protein concentration in tears of office workers. To the best of our knowledge, this is the first study to show the correlation between goblet cell density and MUC5AC protein concentration in tears.

In our second analysis, we observed that goblet cell density was inversely correlated with the LG staining score, which reflects the degree of glycocalyx barrier disruption, but was not correlated with fluorescein staining score, which reflects the degree of epithelial cell defects. These results confirmed a previous report stating that rose Bengal staining score was negatively correlated with goblet cell density in dry eye patients^28^. Consequently, these data suggested that strong LG and rose Bengal staining in conjunctiva may be a valuable approach to estimate goblet cell density reduction without requiring impression cytology.

We found a relationship between cigarette smoking and decreased goblet cell density and MUC5AC secretion in tears. CL use and post-ophthalmic surgery were not significantly related to the decrease in goblet cell density. Moreover, goblet cell density was significantly decreased in current smokers. Some previous reports had shown a significantly decreased number of goblet cells^24^ and higher squamous metaplasia grade in chronic smokers^24^,^30^. However, these reports did not investigate the MUC5AC concentration in tears of smokers^24^. Our data showed that, among office workers who smoked, MUC5AC protein concentration as well as goblet cell density was significantly lower (both p < 0.05) than among non-smokers. Another report had indicated that passive exposure to cigarette smoke was associated with an increase in IL-6 concentration in tears and a decrease in mucosal defense, including a significant downregulation in *MUC5AC* mRNA levels^31^.

Goblet cells have been reported to be susceptible to inflammation^32^ and to a decreased cell number during inflammation status of ocular surface^13^,^14^. These previous reports have proposed that cigarette smoke may induce a reduction in the number of conjunctival goblet cells under inflammatory conditions, resulting in a decrease in MUC5AC protein concentration in tears^13^,^14^,^31^,^32^. Additionally in this study, the pack-years of cigarette smoking, representing a measure of the extent of smoking over a long period of time, was significantly negatively correlated with goblet cell density, as well as with MUC5AC concentration in tears. Previous reports have shown that long-term cigarette smoke exposure and oxidative stress induces a low-grade systemic inflammatory response, as confirmed by numerous population-based studies: elevated levels of C-reactive protein (CRP) and serum level of IL-6, as well as increased counts of WBC have been reported^33^,^34^,^35^. A recent report has shown that pack-years of cigarette smoking were positively correlated with serum IL-6 level in current smokers with 25 pack-years or more of smoking history^36^. Taken together, excessive cigarette smoke exposure over multiple decades may have a direct and indirect impact on the ocular surface, and affect goblet cell density as well as tear MUC5AC concentration.

We did not detect a significant relationship between TBUT values and age. However, Schirmer test value was significantly decreased in subjects aged over 50 years, as compared to those younger than 30 years. Likewise, in previous investigations, the secreted tear volume decreased significantly with increasing age^37^,^38^. Aging induces histopathological changes in the lacrimal gland, and Obata *et al*. have previously shown a significant correlation between age and periductal fibrosis. They suggested that ductal pathologic changes may be an important factor for decreasing tear fluid outflow and/or lacrimal gland dysfunction^39^. In our data, the average Schirmer test value was significantly lower in men than in women. Furthermore, the average age in men was significantly higher than that in women. Our data and those of previous reports^37^,^38^,^39^ suggested that aging may cause a decline in tear secretion. In contrast, no significant difference in mean age was found between current smokers and non-smokers. In a rat model of passive smoking, exposure to mainstream cigarette smoke caused lacrimal gland dysfunction through DNA oxidation by reactive oxygen species, and a reduction in tear secretion^40^. Therefore, our own and previous data^40^ indicated that the impact of cigarette smoking on tear secretion may be independent of the aging effect, but is more likely dependent on oxidative stress.

Moreover, we found that MUC5AC concentration in tears significantly increased with age. As tear secretion decreases with age, the tear exchange rate may slow down and tear proteins tend to be deposited on the ocular surface^38^. In general, MUC5AC as gel-forming mucin is largely responsible for the rheological properties of tear, since MUC5AC has a hydrophilic character resulting from its heavy glycosylation^41^,^42^. Furthermore, MUC5AC is thought to be responsible for the high viscosity of the tear film with lipocaline^43^,^44^. Increased tear MUC5AC concentration due to reduced aqueous secretion, may result in the maintenance of TBUT values with high viscosity of tear in elderly.

There are a few limitations to our study. First, all current smokers in our study were men. Therefore, the decreased goblet cell density and MUC5AC concentration in tears in smokers, as compared to that in non-smokers, might have been affected by the gender and small sample size of the smokers. Second, we never asked current smokers about the timing of cigarette smoking before obtaining the tear wash and impression cytology samples. It is likely that the tear film has a rapid turn-over, and that this timing may have an impact on conditions of the ocular surface. Third, we did not obtain information about the type of ocular surgery. Therefore, we cannot evaluate the possibility of a decrease in goblet cell density early after LASIK^45^, and an increase early after pterygium excision^46^. Fourth, we could not completely assess the influence of other environmental factors, such as low humidity, high temperature, decreased blinking rate, and other indoor pollutants in the subjects’ office rooms.

In summary, our results indicate that chronic smoking may decrease tear secretion, goblet cell density, and MUC5AC protein concentration in tears. Furthermore, goblet cell density in the conjunctiva is positively correlated with MUC5AC concentration in tears, and inversely correlated with glycocalyx barrier disruption, as indicated by LG staining. The findings from this study prove and reinforce previous findings and concepts that cigarette smoking may exacerbate the qualitative and quantitative characteristics of tears, causing a decrease in goblet cell density and decline in lacrimal gland function, and adequate goblet cell density may be important for maintaining an adequate MUC5AC concentration in tears.

## Methods

### Study Design and Subjects

Eighty-eight office workers (age range: 24–62 years) were voluntarily enrolled from a total of 561 subjects among the Osaka study, who agree impression cytology and tear samples collection. The details of the Osaka study have been described previously^47^. The Osaka Study is an epidemiological research that was conducted under the supervision of the Japanese Dry Eye Society to investigate the prevalence of dry eye disease in office workers, who were employed at a company in Osaka, Japan. Thirty-one samples from 176 eyes, which did not meet the qualitative and quantitative criteria for the conjunctiva required for counting goblet cells by means of impression cytology, were excluded. Finally, we included 145 samples from 88 subjects in this study.

The subjects were invited by e-mail to answer the questionnaires, which included questions on general information, such as age, sex, the duration of VDT use, contact lens use (yes or no), history of ocular surgery, smoking, and systemic diseases. Smoking status was evaluated as being a current smoker or not. We also asked about the average number of cigarettes smoked per day, and the number of years of smoking. Pack-years of cigarette smoking were calculated as the average number of cigarettes per day, multiplied by the number of years of smoking. History of systemic diseases (stroke, hypertension, and diabetes mellitus) was determined based on whether any such disease had been diagnosed by physicians. We did not exclude subjects with a history of refractive surgery, since no information was obtained regarding on this matter. This research followed the tenets of the Declaration of Helsinki, and the protocol of this study was approved by the institutional review board of Ryogoku Eye Clinic, Tokyo, Japan. Written informed consent was obtained from all subjects after an explanation of the nature and possible consequences of the study.

### Tear Function Test

Tear stability and quantity were evaluated using two different parameters: TBUT and the Schirmer test value. To determine TBUT, fluorescein vital staining was applied; patients were requested to blink 3 times to ensure adequate mixing of the fluorescein dye with tears. The time interval between the last complete blink and the appearance of the first corneal dark spot was measured using a stopwatch, and the mean of 3 measurements was used for the final analysis. The Schirmer test was performed without anesthesia after all other examinations, using a sterilized Schirmer strip (Whatman No. 41; Showa, Tokyo, Japan). To avoid the influence of conjunctival and corneal staining on the Schirmer test, we proceeded with this test after a 10-min interval.

### Clinical Ocular Surface Evaluation

All subjects were asked not to use a VDT for 2 h before the clinical examinations and sample collections. Briefly, corneal and conjunctival epithelial damage was evaluated using the double-vital staining method^30^. Two microliters of a preservative-free combination of 1% fluorescein (FL) and 1% lissamine green (LG) were instilled into the conjunctival sac using a micropipette. For the purpose of quantifying staining, the eye was divided into 3 equal compartments, representing the nasal conjunctiva, cornea, and temporal conjunctiva, and the maximum staining score for each area was 3 points. The overall epithelial damage was scored on a scale of 0–9 points^48^.

### Tear Sample Collection and Impression Cytology

Briefly, tears were collected from unanesthetized eyes using a micropipette after instillation of 50 μL saline in the cul-de-sac, followed by movement of the eyes to mix the tear fluid content^49^. Individual tear samples were centrifuged for 10 min at 12,000 × gat 20 °C to remove any debris^12^. Immediately after tear collection, impression cytology specimens were obtained after the administration of topical anesthesia using 0.4% oxybuprocaine hydrochloride (Benoxil ophthalmic solution 0.4%; Santen Pharmaceutical Co., LTD., Osaka, Japan)^24^. Cellulose acetate filter paper (Millipore HAWP 304, Bedford, MA, USA) was cut into 3.5 mm × 5 mm strips and applied onto the temporal superior bulbar conjunctiva, pressed gently using a glass rod, and removed. The strips were then carefully transferred into a tube (Eppendorf, Fremont, CA, USA) containing 10% formalin.

### MUC5AC Concentration in Tears

Quantification of the MUC5AC protein in tears was performed using an enzyme-linked immunoassay (ELISA), using our previously reported method^12^. Firstly, protein concentration in tears was determined (1:100 dilution of sample in a micro-plate) with a protein assay reagent kit (MicroBCA; Pierce, Rockford, IL). Next, the concentration of the secreted mucin MUC5AC in the tear samples was quantified using an ELISA kit (E90756Hu; USCN Life Science, Huston, TX). All samples were analyzed according to the manufacturer’s guidelines. Absorbance was measured at 450 nm, and the standard solutions in the kit were recombinant human MUC5AC. The MUC5AC concentration was normalized to the tear total protein content and expressed as MUC5AC protein (nanograms) per tear total protein (milligrams).

### Measurements of Goblet Cell Density in the Conjunctiva

The impression cytology specimens were fixed with formalin, and stained using the periodic acid-Schiff technique. Samples were oxidized in 0.5% periodic acid solution for 5 min, washed with distilled water, and placed in Schiff reagent for 10 min. After counterstaining with hematoxylin solution for 30 s and then washing, the membranes were dehydrated in ascending grades of ethanol, and then with xylene, and finally mounted with coverslips. Goblet cells were counted in 3 non-overlapping areas in each specimen, chosen at random with the help of a calibrated grid. The goblet cell densities were reported as cells per square millimeter, with standard deviations^24^,^50^. A researcher who was masked to the identity of the samples evaluated the specimens for goblet cell counts.

### Analysis

All data are presented as mean ± standard deviation. Depending on the distribution of individual data, the significance of differences in each ocular surface evaluation factor (TBUT, Schirmer test value, LG staining score, FL staining score, goblet cell density, and MUC5AC concentration) and age groups, CL use, current smoking status, and history of ophthalmic surgery was determined by the Steel test or the Kruskal−Wallis test. Multiple regression analysis was performed to study the correlation between age, contact lens use, smoking status, VDT use (in hours), and goblet cell density or tear MUC5AC concentration. In addition, ranking values of goblet cell density or tear MUC5AC concentration were used for multiple regression analysis. The age (grouped per 10 years), CL use, current smoking status, and VDT use (>8 h or ≤8 h per day) were used as independent variables. Correlation between goblet cell density and other ocular surface evaluation factors, and the correlation of pack-year of smoking with goblet cell density and MUC5AC protein concentration in tears was analyzed using Spearman’s rank correlation test. The P values were 2-sided and the level of significance was set as 5% (P < 0.05). All statistical analyses were performed using SAS, version 9.2 (SAS Institute Inc., Cary, NC).

## Additional Information

**How to cite this article**: Uchino, Y. *et al*. Impact of Cigarette Smoking on Tear Function and Correlation between Conjunctival Goblet Cells and Tear MUC5AC Concentration in Office Workers. *Sci. Rep.* **6**, 27699; doi: 10.1038/srep27699 (2016).

## Figures and Tables

**Table 1:** Characteristics of the study population.

		Men	Women	Total
		N = 56	N = 32	N = 88
		N (%) or Mean ± SD	N (%) or Mean ± SD	N (%) or Mean ± SD
Age (years)	20–29	6 (10.7)	7 (21.9)	13 (14.8)
	30–39	12 (21.4)	10 (31.3)	22 (25.0)
	40–49	18 (32.1)	13 (40.6)	31 (35.2)
	50+	20 (35.7)	2 (6.3)	22 (25.0)
	Total	44.3 ± 10.1	37.4 ± 7.7	41.8 ± 9.9
VDT working time (h)		6.3 ± 1.9	7.5 ± 1.7	6.8 ± 1.9
Current smoker		16 (28.6)	0 (0)	16 (18.2)
Contact lens user		12 (21.4)	19 (59.4)	31 (35.2)
Post-ophthalmic surgery		7 (12.5)	2 (6.3)	9 (10.2)
Past/current history of certain common systemic diseases,				
Stroke		1 (1.8)	0 (0)	1 (1.1)
Hypertension		5 (8.9)	0 (0)	5 (5.7)
Diabetes mellitus		2 (3.6)	0 (0)	2 (2.3)

N = number of subjects enrolled, VDT = visual display terminal.

**Table 2:** Average tear condition evaluation factors in categorized groups.

		N (%)	TBUT (s)			Schirmer test value (mm)		
			Mean ± SD	Kruskal-Wallis P value	Steel P value	Mean ± SD	Kruskal-Wallis P value	Steel P value
Age (years)	(1) 20–29	21 (14.5)	4.9 ± 3.0	0.593	1:2 P = 0.968 1:3 P = 0.420 1:4 P = 0.770	24.6 ± 11.1	0.001	1:2 P = 0.124 1:3 P = 0.467 1:4 P = 0.001
	(2) 30–39	34 (23.4)	4.6 ± 2.8			17.4 ± 12.4		
	(3) 40–49	47 (32.4)	4.0 ± 2.6			20.5 ± 12.0		
	(4) 50+	43 (29.7)	4.2 ± 2.7			12.5 ± 9.1		
	Total	145 (100.0)	4.3 ± 2.7	–	–	18.0 ± 11.8	–	–
Sex	Men	95 (65.5)	4.5 ± 3.0	0.406	–	16.6 ± 11.8	0.047	–
	Women	50 (34.5)	3.9 ± 2.1			20.7 ± 11.5		
Contact Lens	Non user	97 (66.9)	4.2 ± 2.8	0.144	–	16.5 ± 11.7	0.041	–
	User	48 (33.1)	4.6 ± 2.5			20.9 ± 11.6		
Current Smoker	No	119 (82.1)	4.2 ± 2.5	0.601	–	19.0 ± 11.7	0.016	–
	Yes	26 (17.9)	4.6 ± 3.5			13.3 ± 11.5		
Post Ophthalmic Surgery	No	128 (88.3)	4.4 ± 2.7	0.287	–	18.6 ± 11.6	0.050	–
	Yes	17 (11.7)	3.7 ± 2.5			13.5 ± 12.4		

N = number of samples enrolled, TBUT = tear break up time.

**Table 3:** Average goblet cell density in the bulbar conjunctiva and tear MUC5AC concentration in categorized groups.

		N (%)	Goblet Cell Density (cells/mm^2^)			MUC5AC Concentration (ng/mg)		
			Mean ± SD	Kruskal-Wallis P value	Steel P value	Mean ± SD	Kruskal-Wallis P value	Steel P value
Age	(1) 20–29	21 (14.5)	81.0 ± 57.4	0.387	1:2 P = 0.984 1:3 P = 0.954 1:4 P = 0.550	4.4 ± 3.3	0.007	1:2 P = 0.956 1:3 P = 0.915 1:4 P = 0.016
	(2) 30–39	34 (23.4)	93.5 ± 109.5			4.8 ± 3.6		
	(3) 40–49	47 (32.4)	84.1 ± 78.1			5.8 ± 6.2		
	(4) 50+	43 (29.7)	124.8 ± 122.0			11.4 ± 13.1		
	Total	145 (100.0)	97.9 ± 99.1	–	–	7.0 ± 8.7	–	–
Sex	Men	95 (65.5)	93.1 ± 93.3	0.333	–	6.8 ± 8.8	0.891	–
	Women	50 (34.5)	107.2 ± 109.7			7.4 ± 8.6		
Contact Lens	Non user	97 (66.9)	97.9 ± 94.4	0.679	–	6.9 ± 8.7	0.705	–
	User	48 (33.1)	98.1 ± 109.1			7.2 ± 8.8		
Current Smoker	No	119 (82.1)	106.7 ± 106.5	0.036	–	7.6 ± 9.3	0.024	–
	Yes	26 (17.9)	57.6 ± 30.8			4.1 ± 3.9		
Post Ophthalmic Surgery	No	128 (88.3)	94.3 ± 94.0	0.694	–	6.1 ± 6.5	0.119	–
	Yes	17 (11.7)	125.1 ± 131.9			13.6 ± 17.0		

N = number of samples enrolled.

**Table 4:** The multiple regression analysis for goblet cell density and tear MUC5AC concentration using age, contact lens, smoking status, and VDT use as variables.

Variables		Goblet Cell Density (cells /mm^2^)		Tear MUC5AC Concentration (ng/mg)	
		Parameter Estimate (95% CI)	P value	Parameter Estimate (95% CI)	P value
Age	10-year increase	3.83 (−3.42 to 11.08)	0.298	11.44 (4.42 to 18.45)	0.002
Contact Lens	User	−4.17 (−20.95 to 12.61)	0.624	2.80 (−13.42 to 19.03)	0.733
Smoking Status	Current	−20.72 (−38.86 to −2.58)	0.026	−21.97 (−39.51 to −4.43)	0.015
VDT use	8 h or more	5.23 (−12.00 to 22.47)	0.549	4.39 (−12.27 to 21.05)	0.603

CI = confidence interval.

**Table 5:** Correlation of goblet cell density with MUC5AC concentration in tears, age, VDT working time, and ocular surface evaluation factors.

Goblet Cell Density vs.	Spearman’s rank correlation coefficient	P value
Tear MUC5AC protein	0.181	0.030
Ocular Surface Evaluation Factors		
Schirmer test value	0.002	0.981
TBUT	0.069	0.406
LG staining score	−0.185	0.026
FL staining score	−0.052	0.536

VDT = visual display terminal, TBUT = tear break up time, LG = lissamine green, FL = fluorescein.
